# Virulence and Genomic Feature of Multidrug Resistant *Campylobacter jejuni* Isolated from Broiler Chicken

**DOI:** 10.3389/fmicb.2016.01605

**Published:** 2016-10-14

**Authors:** Haihong Hao, Ni Ren, Jing Han, Steven L. Foley, Zahid Iqbal, Guyue Cheng, Xiuhua Kuang, Jie Liu, Zhenli Liu, Menghong Dai, Yulian Wang, Zonghui Yuan

**Affiliations:** ^1^National Reference Laboratory of Veterinary Drug Residues (HZAU) and MOA Key Laboratory for Detection of Veterinary Drug ResiduesWuhan, China; ^2^MOA Laboratory for Risk Assessment of Quality and Safety of Livestock and Poultry ProductsWuhan, China; ^3^Hubei Collaborative Innovation Center for Animal Nutrition and Feed Safety, Huazhong Agricultural UniversityWuhan, China; ^4^Division of Microbiology, FDA, National Center for Toxicological ResearchJefferson, AR, USA

**Keywords:** *Campylobacter jejuni*, broiler chicken, multidrug resistance, virulence, *de novo* genome sequencing

## Abstract

The aim of this study was to reveal the molecular mechanism involved in multidrug resistance and virulence of *Campylobacter jejuni* isolated from broiler chickens. The virulence of six multidrug resistant *C. jejuni* was determined by *in vitro* and *in vivo* methods. The *de novo* whole genome sequencing technology and molecular biology methods were used to analyze the genomic features associated with the multidrug resistance and virulence of a selected isolate (*C. jejuni* 1655). The comparative genomic analyses revealed a large number of single nucleotide polymorphisms, deletions, rearrangements, and inversions in *C. jejuni* 1655 compared to reference *C. jejuni* genomes. The co-emergence of Thr-86-Ile mutation in *gyrA* gene, A2075G mutation in 23S rRNA gene, *tetO, aphA* and *aadE* genes and pTet plasmid in *C. jejuni* 1655 contributed its multidrug resistance to fluoroquinolones, macrolides, tetracycline, and aminoglycosides. The combination of multiple virulence genes may work together to confer the relative higher virulence in *C. jejuni* 1655. The co-existence of mobile gene elements (e.g., pTet) and CRISPR-Cas system in *C. jejuni* 1655 may play an important role in the gene transfer and immune defense. The present study provides basic information of phenotypic and genomic features of *C. jejuni* 1655, a strain recently isolated from a chicken displaying multidrug resistance and relatively high level of virulence.

## Introduction

*Campylobacter jejuni* is one of the most important foodborne pathogens worldwide (Abril et al., 2010). The macrolides (mainly erythromycin and azithromycin) and fluoroquinolones are the empirical drugs of choice for treatment of campylobacteriosis (Allos, 2001). Other antimicrobial agents, including gentamicin, meropenem, and clindamycin are alternative therapies (Iovine et al., 2008).

The National Antimicrobial Resistance Monitoring System (NARMS) in the USA and Danish Integrated Antimicrobial Resistance Monitoring and Research Program (DANMAP) data showed that multidrug resistance in *C. jejuni* has been very rare (0.3-0.7%) from retail chicken meat (DANMAP, 2014; NARMS, 2015). Multidrug resistant *C. jejuni* have been isolated from chicken farms in China recently (Chen et al., 2010; Hao et al., 2015; Wang et al., 2016). The multidrug resistant *C. jejuni* in chicken may greatly threaten food safety and human public health, therefore it is important to investigate the virulence potential and mechanism involved in multidrug resistance and virulence of *C. jejuni*.

Although genomes of many human-source *C. jejuni* (e.g., NCTC11168, 81–176, 260.94, HB93-13, CF93-6, and 269.97) have been sequenced, only three genomes of *C. jejuni* (305, RM1221, and 81-176-DRH212) from poultry have been obtained (Parkhill et al., 2000; Fouts et al., 2005; Hofreuter et al., 2006). *C. jejuni* RM1221 (ATCC BAA-1032) is a chicken isolate with unique lipoolgosaccharide and ability to colonize chicken skin (Fouts et al., 2005). *C. jejuni* 305 is a turkey isolate with stress tolerance (Takamiya et al., 2011). *C. jejuni* 81–176-DRH212 is a *C. jejuni* 81–176 variant with enhanced fitness in the chicken gastrointestinal tract (Johnson et al., 2014). The genome sequences of broiler *C. jejuni* isolates with multidrug resistance and high virulence have not been published previously. In order to investigate the mechanisms involved in the multidrug resistance and increased virulence in *C. jejuni* isolated from broiler chickens in China, the genomic profile of a *C. jejuni* isolate with relative higher virulence and multidrug resistance was determined by the *de novo* sequencing technology in the present study.

## Materials and methods

### Isolation and identification of chicken *C. jejuni*

Six strains (1442, 1447, 1614, 1622, 1655, and 1685) were isolated from caecum of healthy broiler chicken in the chicken farms located in the center of China in 2013. These farms have a long history of usage of different antimicrobial drug for prevention and treatment chicken disease. These strains were confirmed as *C. jejuni* by classic biochemical test and PCR amplification of the 16S rRNA, *mapA* and *vs1* genes (Stucki et al., 1995; Hao et al., 2013, 2015). The *C. jejuni* isolates were grown on Mueller-Hinton (MH) agar supplemented with 5% sheep blood at 42°C under microaerobic conditions (5% O_2_, 10% CO_2_, and 85% N_2_) for 24–48 h.

### Antimicrobial susceptibility test

The minimum inhibitory concentrations (MICs) of erythromycin (ERY), tylosin (TYL), ciprofloxacin (CIP), enrofloxacin (ENR), doxycycline (DOX), tetracycline (TET), amikacin (AMK), and gentamicin (GEN) were determined using the agar dilution method as recommended by the Clinical and Laboratory Standards Institute (CLSI) M31-A3 guidelines (CLSI., 2008). The *C. jejuni* ATCC33560 was used as quality control for the MIC determination.

### Preliminary pathogenicity test

Newly hatched broiler chickens were purchased from Zhengda Limited Company (Wuhan, China). Prior to the experiment, all the chickens tested negative for *C. jejuni* strains by *C. jejuni* isolation and identification methods. The chickens were randomly divided into 8 groups with 7 chickens in each group. The chickens in groups 1–7 were administrated once with 5 × 10^5^ CFU of each *C. jejuni* strain (1442, 1447, 1614, 1622, 1655, 1685, and RM1221) by oral injection, respectively. The eight group served as the negative control with no bacterial infection. The clinical symptoms and mortality of the chickens in each group was observed daily. The colonization rate of each *C. jejuni* strain was determined using selective medium containing 32 αg/mL erythromycin.

### Cytotoxin assay

The murine macrophage RAW264.7 cell line was used to evaluate the cytotoxic effects of the six *C. jejuni* isolates. The tissue culture cytotoxin assay was carried out as previously described (Guerrant et al., 1987) with some modification. Briefly, *C. jejuni* was treated by 2000 μg/ml polymyxin B for 1 h at 37°C (Ashkenazi and Cleary, 1990) and centrifuged at 8000 rpm for 20 min. The supernatant were filtered through 0.22 μm filter. Purified shiga toxin (Chinese Center for Disease Control and Prevention) and 0.3% Triton X-100-PBS were used as positive controls, and polymyxin B-treated broth and polymyxin B-PBS (phosphate-buffered saline) were used as negative controls. Suspensions of murine macrophage RAW264.7 cells (100 μl; 5 × 10^5^ cells) were placed in 96-well flat-bottom microtiter plates and allowed to adhere for 1 to 3 h to form the tissue culture monolayers. Serial twofold dilutions of the prepared *C. jejuni* filtrates were added to the tissue culture monolayers in 100 μl volumes. The monolayers were incubated with the filtrates at 37°C in 5% CO_2_ for 24 h. The monolayers were then examined by phase-contrast microscopy for the percentage of cells rounded. Cell death was determined by trypan blue dye uptake (A540 value) and correlated with cell morphology after Giemsa staining. The index of cytotoxin effect was calculated by the formula.

$$100\times \left[1-\frac{A540(test)-A540(positive)}{A540(negative)-A540(positive)}\right].$$

### Biofilm assays

To measure the biofilm formation of the six *C. jejuni* strains, crystal violet staining was used as described previously for *C. jejuni* and other bacteria (Asakura et al., 2007; Fields and Thompson, 2008; McLennan et al., 2008; Reuter et al., 2010) with some modification. Briefly, the 200 αl of *C. jejuni* fresh culture (OD_600_ = 0.05) was added to 96-well flat-bottom microtiter plates. Plates were incubated without shaking at 42°C under microaerobic conditions for 24, 48, and 72 h. For crystal violet staining, each well was washed with PBS three times to remove the planktonic cells. The 200 αl of methanol were added and incubated for 15 min and then dried at room temperature. Then 200 αl of 0.1% Hucker crystal violet solution were added, and the plates were incubated at room temperature for 5 min. Unbound crystal violet was washed off with PBS, and the plates were dried at 60°C. Bound crystal violet was dissolved in 30% glacial acetic acid for 10 min. The absorbance was determined using a plate reader at 570 nm. The wells with sterile medium were used as blank control. The blank corrected absorbance values of *C. jejuni* strains were used for reporting biofilm production. Assays were repeated at least three times with three technical replicates.

### LD_50_ determination

Based on the results from the preliminary pathogenicity experiments with chickens, cytotoxin testing and biofilm assays, three *C. jejuni* isolates (1442, 1622, and 1655) were selected to further determine their median lethal dose (LD_50_) using 2 days old chicken orally or intraperitoneally infected with a range of concentration (10^5^–10^7^ CFU) of each *C. jejuni* isolate. All the chickens tested negative for *C. jejuni* prior to inoculation using the *C. jejuni* isolation and identification methods described above. The chickens were randomly divided into six groups with 35 chickens in each group. The chickens in each group were orally or intraperitoneally administrated with 0, 10^5^, 10^6^, or 10^7^ CFU of the *C. jejuni* isolates, respectively. Seven chickens were inoculated with each of the dilution of the test strains.

### Adhesion and invasion assay

The adhesion and invasion of these three *C. jejuni* strains (1442, 1622, and 1655) was determined as previously described (Almofti et al., 2011a). Briefly, 5.0 × 10^7^ CFU/mL of the *C. jejuni* strain was used to inoculated the monolayers of macrophage RAW264.7 cells at multiplicity of infection (MOI) of 100. The infected monolayers were incubated for 3 h to allow the occurrence of adhesion and invasion. For determination of the total number of adherent and internalized bacteria, the monolayers were washed three times with Dulbecco's Modified Eagle's Medium (DMEM) without antibiotic to remove the extracellular unbound bacteria. The monolayers were then lysed to release the intracellular bacteria. For determination of the invading bacteria, the monolayers were washed twice with aspirated DMEM medium and 100 mg/ml gentamicin was added for 1 h to kill the extracellular and bound bacteria. The monolayers were then washed three times and lysed to release intracellular bacteria. The number of adherent bacteria was obtained by subtraction of the internalized bacteria number from total number of adherent and internalized bacteria.

### Intracellular survivability assay

To determine the intracellular survivability of *C. jejuni* within macrophage RAW264.7, the invasion period of each *C. jejuni* strain (1442, 1622, and 1655) was extended to 3, 6, 10, 16, 24, 48, and 72 h post-infection. Cells were washed and lysed as previously described in the adhesion and invasion assay. The surviving intracellular bacteria were enumerated by plating serial dilutions on blood agar and counting the resultant colonies.

### Motility test

To determine the migration of the three selected strains (1442, 1622, and 1655), a fresh culture of *C. jejuni* strain was inoculated into MH broth and cultured to their logarithmic growth phase. Then 3 αl of bacteria with consistent concentration were stabbed into 0.4 % MH agar. Plates were incubated at 42°C under microaerophilic conditions for 24 h and the motility was scored by measuring the diameter of the growth in each plate. The experiment was performed in triplicate.

### *De novo* whole-genome sequencing of *C. jejuni* 1655

Total genomic DNA of *C. jejuni* 1655 was extracted using the TIANamp Bacteria DNA kit according to the manufacturer's protocol. The genomic DNA was sent to Shanghai Biotechnology Corporation for the *de novo* whole genome sequencing performed in a HiSeq 2500 platform using a multiplexed, 2 × 100 nucleotide paired end approach (Illumina). The sequence analysis and assembly were carried out using CLC Genomics Workbench (ver. 6.0., Qiagen). Prediction and identification of the main components of the genome, including open reading frames (ORFs), tRNA, rRNA, ncRNA, CRISPR-Cas system, and repeat elements, were conducted using software of Glimmer 3.02 and some other online tools.

The nucleotides and predicted proteins of 1655 were compared with previously sequenced *C. jejuni* genomes in GenBank using updated nt and nr databases with BLAST software. The genomes used as reference sequences for the comparative genomic analysis were *C. jejuni* 81–176, *C. jejuni* NCTC11168, *C. jejuni* RM1221. The comparative genomic analysis was performed using BLASTN, BLASTX, Mummer and Quast. The sequence data from *C. jejuni* 1655 was compared to the results of antimicrobial resistance and virulence to identify genetic factors that correspond to the observed mechanisms.

### Nucleotide sequence accession numbers

The whole genome sequence of *C. jejuni* 1655 has been deposited at DDBJ/ENA/GenBank under the accession MDDM00000000.

### Ethics statement

All the experimental procedure in this study was performed according to the guidelines of the committee on the use and care of the laboratory animals in Hubei province China. The study was approved by Animal Ethics Committee of Huazhong Agricultural University (hzauch 2014-002) and the Animal Care Center, Hubei Science and Technology Agency in China (SYXK 2013–0044). These experiments were in line with national regulations about animal welfare ethics. All the animals were monitored throughout the study for any adverse effect signs. All efforts were made to minimize suffering of animals.

## Results

### Minimum inhibitory concentration (MIC)

The minimum inhibitory concentrations (MICs) of different antimicrobial agents against the 6 *C. jejuni* isolates (1442, 1447, 1614, 1622, 1655, and 1685) were presented in Table 1. Each isolate exhibited resistance to all the tested drugs, while three (1622, 1655, and 1685) showed higher-level MICs to erythromycin and gentamicin than the others.

**Table 1:** **MICs of different antimicrobial agents against six *C. jejuni* isolates (μg/mL)**.

**Strains**	**MICs of different antimicrobial agents**							
	**ERY**	**TYL**	**ENR**	**CIP**	**TET**	**DOX**	**GEN**	**AMK**
1442	128	>256	64	128	128	32	64	16
1447	64	>256	32	8	128	32	1	8
1614	256	>256	0.25	16	64	64	>256	4
1622	512	>256	256	128	>256	32	>256	64
1655	512	>256	32	64	256	32	>256	8
1685	512	>256	16	32	64	32	>256	16
RM1221	0.5	2.0	0.5	0.125	0.5	0.25	0.5	0.5

ERY, Erythromycin; TYL, Tylosin; ENR, Enrofloxacin; CIP, Ciprofloxacin; TET, tetracycline; DOX, Doxycycline; GEN, Gentamicin; AMK, Amikacin.

### Pathogenicity of *C. jejuni* isolates on chicken

After oral infection with 10^5^ CFU of the *C. jejuni* isolates, no clinical change was observed in two groups infected with 1442 or 1447, while the other four isolates (1614, 1622, 1655, and 1685) and RM1221 caused different degrees of diarrhea and bloody stools, respectively. The clinical signs of three strains (1614, 1685, and RM 1221) were recovered at day 4, while serious diarrhea caused by 1622 and 1655 was sustained for over 8 and 10 days, respectively (Table S1). Therefore, 1655 exhibited relative higher pathogenicity on chickens than RM1221 and other five *C. jejuni* isolates. Within 15 days, the mortality of all the strains was 0% following oral inoculation of 2 days-old chicken.

### Colonization of *C. jejuni* isolates in chicken

**Figure 1:**
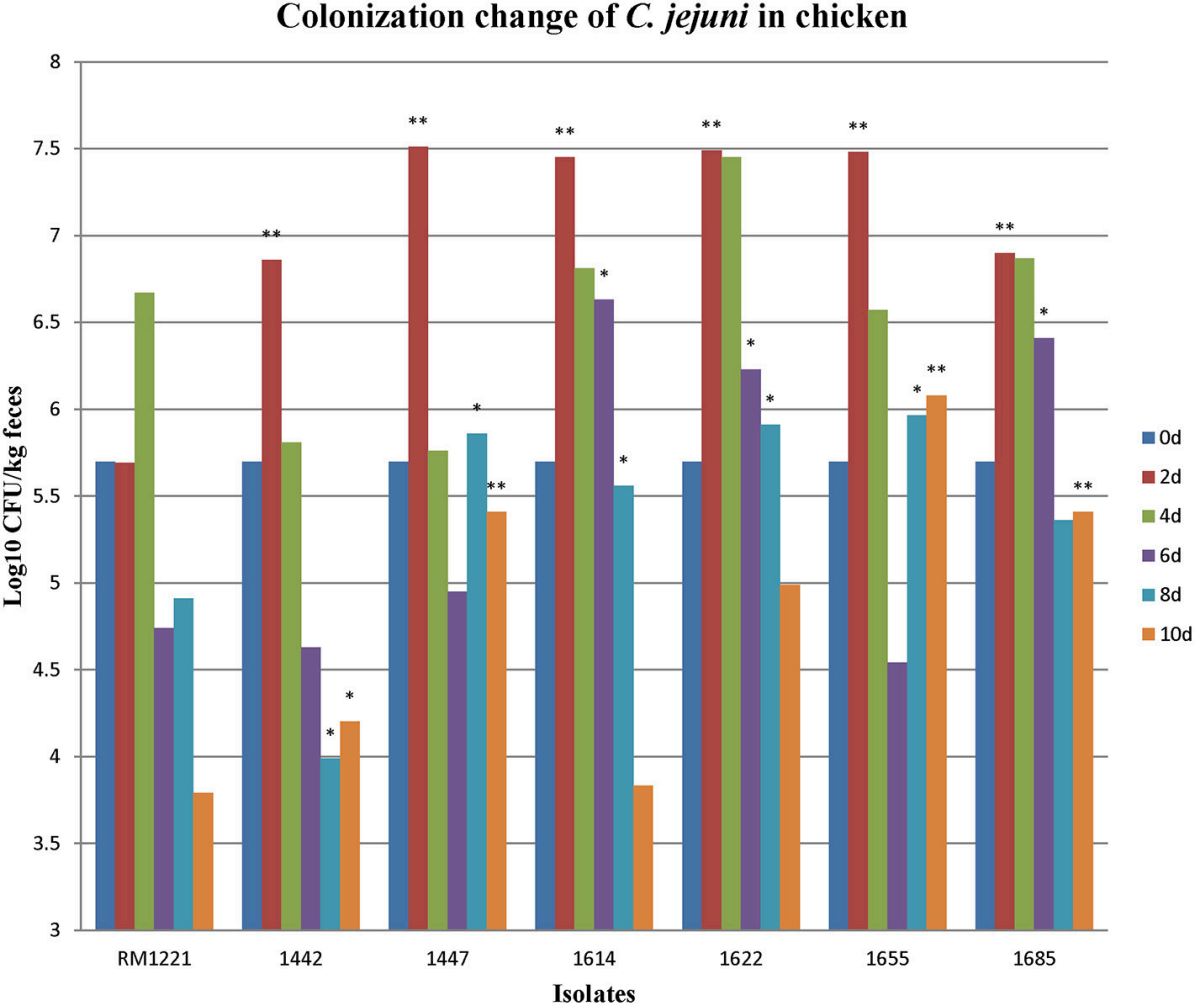
**Colonization change of *C. jejuni* isolates in chicken after inoculation**. The Y-axis is the number (Log_10_ CFU/kg fece) of each strain colonizing the birds at different days. The asterisk (^*^) and (^**^) represent statistical significant difference with *P* ≤ 0.05 and *P* ≤ 0.01 comparing with *C. jejuni* RM1221, respectively.

After oral administration with 5 × 10^5^ CFU of the *C. jejuni* isolates to 2-days old chickens, all the strains could colonize chickens' intestinal tract. Fecal samples were obtained at different time points (2, 4, 6, 8, and 10 days). RM1221 colonized at concentration of 10^5^–10^7^CFU/kg feces at day 2 and day 4, but the concentration was reduced to less than 10^4^ CFU/feces at day 10 (Figure 1). The concentrations of five isolates (1442, 1447, 1622, 1655, and 1685) were higher than 10^4^ CFU/kg feces within the ten days (Figure 1). Comparing with the colonization of RM1221, three isolates (1622, 1655, and 1685) exhibited significantly stronger colonization (Figure 1).

### Cytotoxin of the six *C. jejuni* isolates in macrophage cells

**Figure 2:**
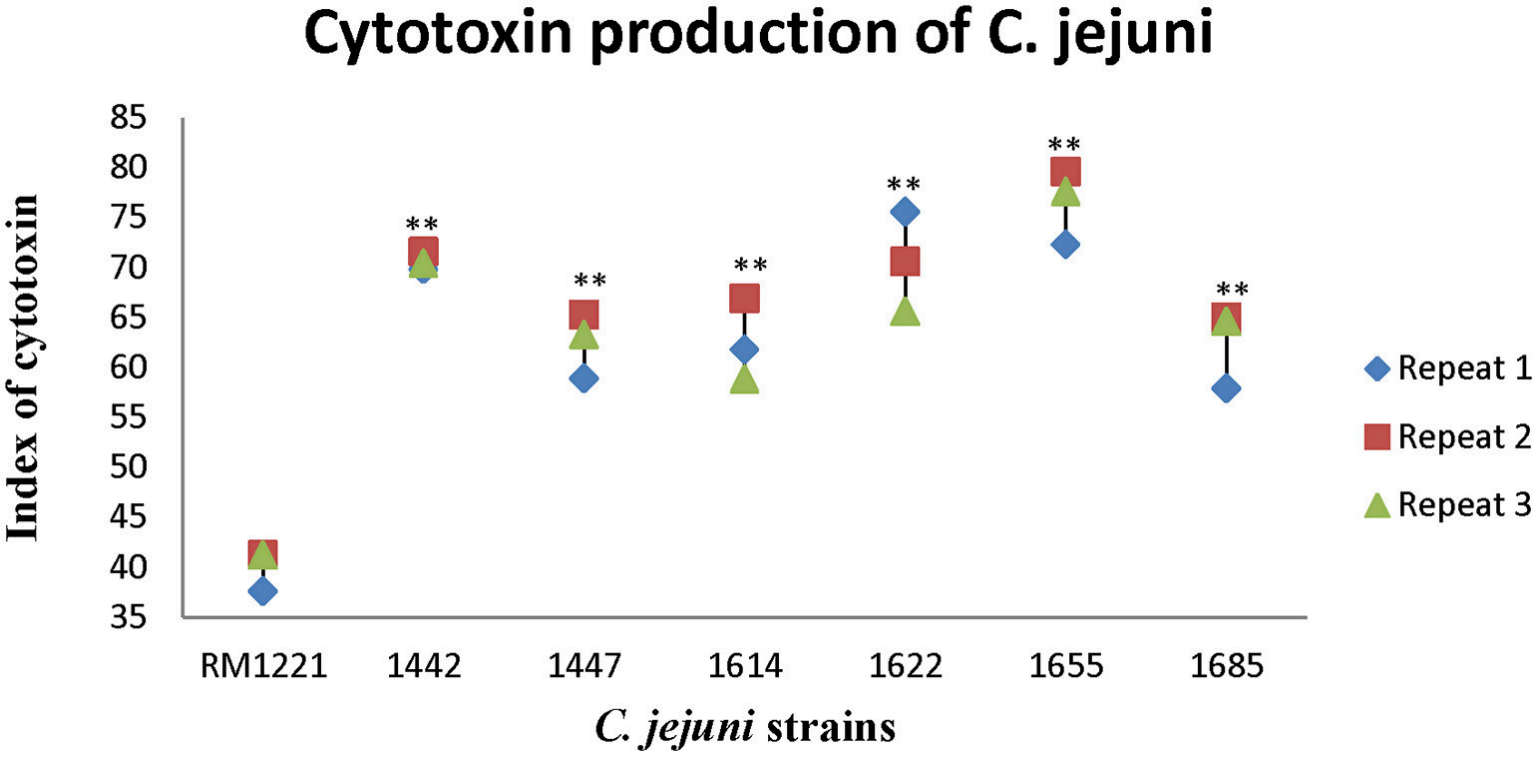
**Cytotoxic production of *C. jejuni* isolates in murine macrophage RAW264.7 cell**. The Y-axis is the index of cytotoxic production of each strain. The results were obtained from three independent repeats. The asterisk (^*^) and (^**^) represent statistical significant difference with *P* ≤ 0.05 and *P* ≤ 0.01 comparing with *C. jejuni* RM1221, respectively.

Figure 2 showed the killing effect (index) of cytotoxin released by six strains (1442, 1447, 1614, 1622, 1655, and 1685) on murine macrophage RAW264.7. The killing effect of cytotoxin from all the strains was lower than 80%. However, compared to RM 1221, significant differences of the index of cytotoxin were observed in these *C. jejuni* strains. Among them, 1442, 1622, and 1655 seemed to release more cytotoxin and therefore exhibited higher killing effect on murine macrophage RAW264.7.

### Biofilm formation of the six *C. jejuni* isolates

**Figure 3:**
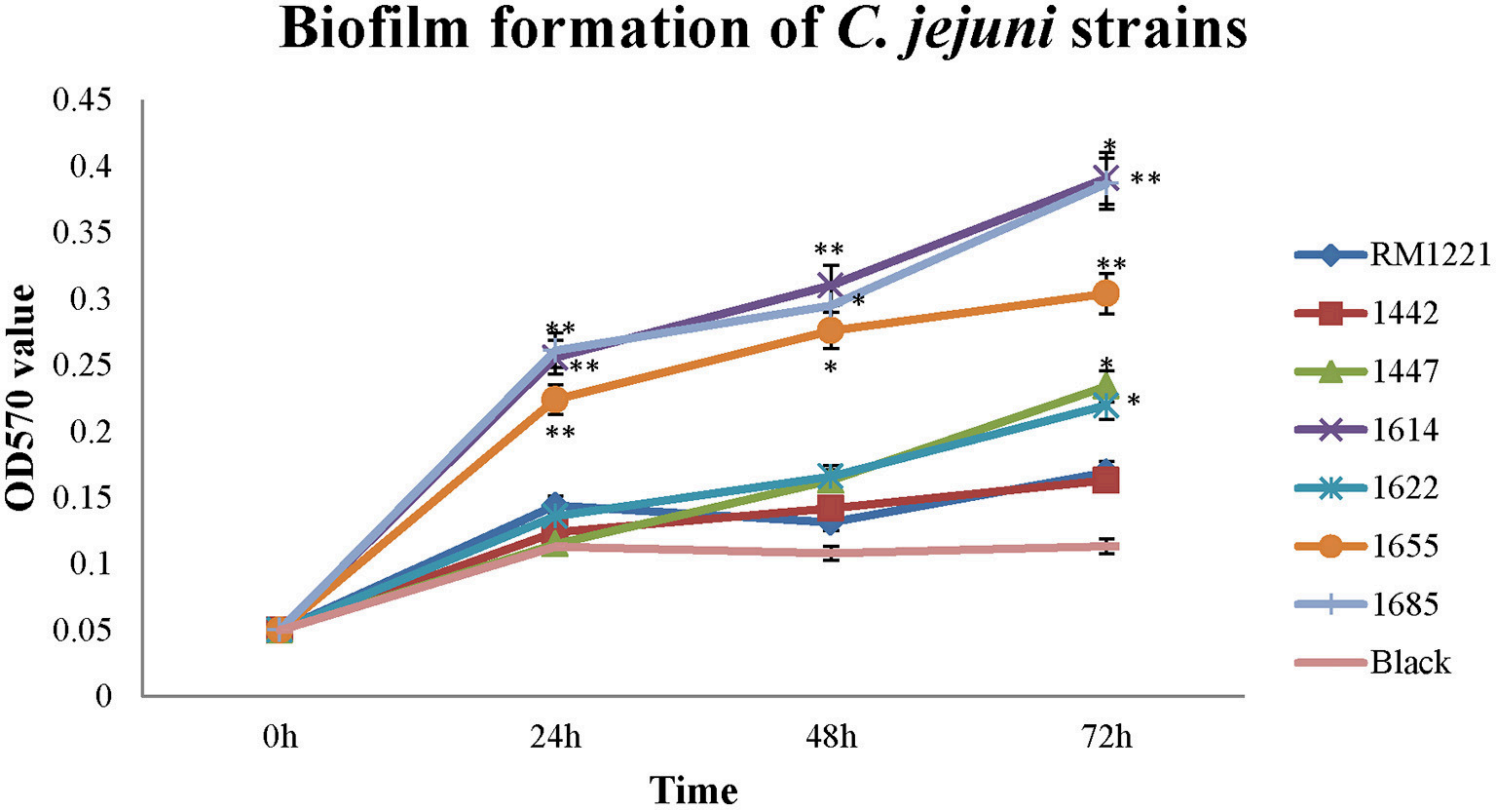
**Biofilm formation of *C. jejuni* isolates at different time points under ordinary conditions**. The Y-axis is the OD_570_ value of crystal violet in biofilm. The results were obtained from three independent experiments. The asterisk (^*^) and (^**^) represent statistical significant difference with *P* ≤ 0.05 and *P* ≤ 0.01 comparing with *C. jejuni* RM1221, respectively.

Different level of biofilm formation was observed in each *C. jejuni* strain (Figure 3). It seemed that the level of biofilm formation had positive correlation with the incubation time. The highest level of biofilm formation was occurred after 72 h incubation (Figure 3). Compared to RM1221, a significant difference of biofilm formation was observed in three isolates (1614, 1655, and 1685) after each incubation time, indicating that these three strains may have stronger ability to form biofilm (Figure 3).

### LD_50_ of the three selected *C. jejuni* isolates on chicken

From the preliminary pathogenicity testing on chicken, cytotoxin testing and biofilm assays, three isolates (1442, 1622, and 1655) were selected for LD_50_determination. As the result shown in Table 2, different fatality rates were observed in different groups. After oral infection, the highest concentration (3.70 × 10^7^ CFU) of 1442 and 1622 could cause mortality rate of 0% (0/7) and 28.6% (2/7) respectively, while 3.7 × 10^7^ CFU of 1655 cause fatality rate of 55.6% (4/7). For intraperitoneal infection, 3.7 × 10^6^CFU of 1442 and 1622 only leaded to one chicken death, while the lowest concentration (3.7 × 10^5^ CFU) of 1655 leaded to 100% death of chickens. The 1655 exhibited highest pathogenicity on chicken with the calculated LD_50_ of 8.45 × 10^7^ CFU for oral infection and LD_50_ of less than 3.7 × 10^5^ CFU for Intraperitoneal infection (Table 2).

**Table 2:** **LD_50_ of the three multidrug resistant strains on 2 days old chickens**.

**Infection route**	**Strains**	**Infection dose of (CFU)**	**Number of chickens**	**Number of Dead chicken**	**LD_50_**
Oral infection	1442	3.70 × 10^7^	7	0	NA
		3.70 × 10^6^	7	0	
		3.70 × 10^5^	7	0	
		0	7	0	
	1622	3.70 × 10^7^	7	2	NA
		3.70 × 10^6^	7	0	
		3.70 × 10^5^	7	0	
		0	7	0	
	1655	3.70 × 10^7^	7	4	8.45 × 10^7^
		3.70 × 106	7	1	
		3.70 × 10^5^	7	1	
		0	7	0	
Intraperitoneal infection	1442	3.70 × 10^7^	7	2	NA
		3.70 × 10^6^	7	1	
		3.70 × 10^5^	7	0	
		0	7	0	
	1622	3.70 × 10^7^	7	1	NA
		3.70 × 10^6^	7	1	
		3.70 × 10^5^	7	0	
		0	7	0	
	1655	3.70 × 10^7^	7	7	<3.70 × 10^5^
		3.70 × 10^6^	7	7	
		3.70 × 10^5^	7	7	
		0	7	0	

### Adhesion and invasion of *C. jejuni* on macrophage RAW264.7

**Figure 4:**
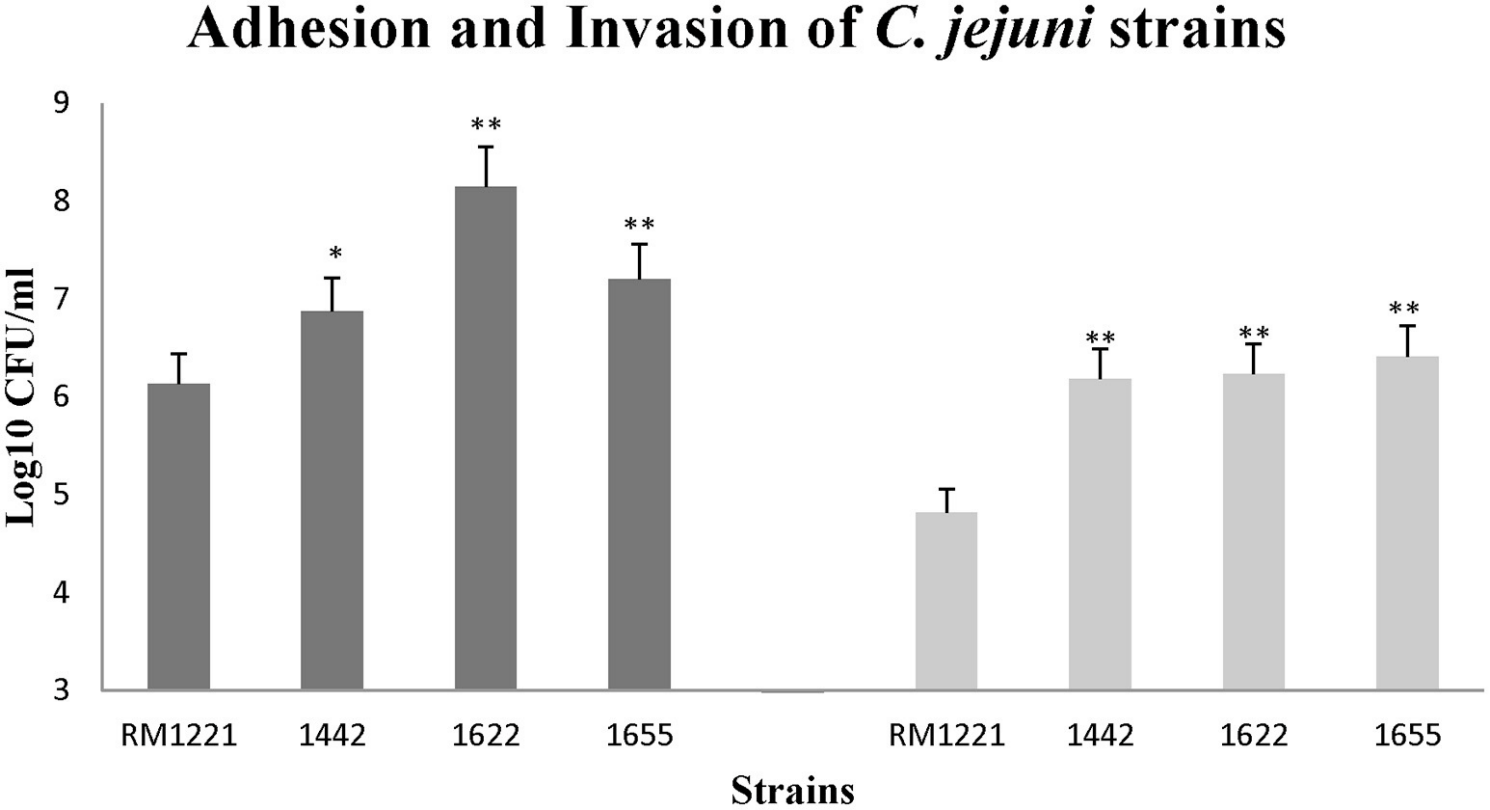
**Adhesion (left) and invasion (right) of *C. jejuni* strains in macrophage RAW264.7 cell**. The Y-axis is the mean of log10 CFU/mL of each strain in the cells. The results were obtained from three independent experiments. The asterisk (^*^) and (^**^) represent statistical significant difference with *P* ≤ 0.05 and *P* ≤ 0.01 comparing with *C. jejuni* RM1221, respectively.

The three isolates (1442, 1622, and 1655) exhibited remarkable higher adhesion and invasion to macrophage cell RAW264.7 than RM1221 (Figure 4). The significant change of adhesion to macrophage cells (*P* < 0.01) was observed in 1622 and 1655. However, there was no significant difference in the adhesion and invasion between three selected isolates (1442, 1622, and 1655).

### Intracellular survivability of *C. jejuni* in macrophage RAW264.7

**Figure 5:**
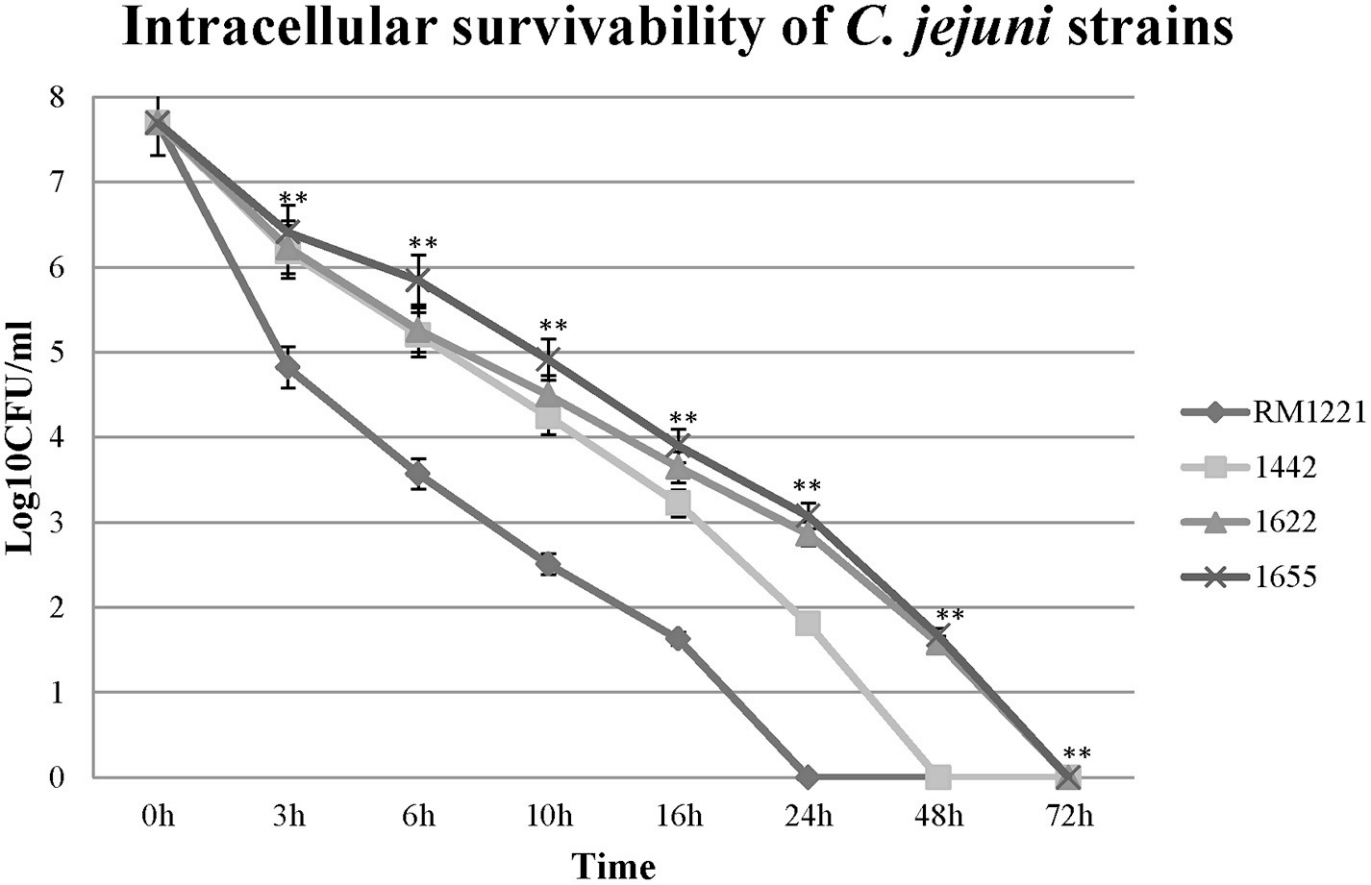
**Intra-macrophage survival assay of three selected *C. jejuni* strains in macrophage RAW264.7 cell**. The Y-axis is the mean of log10 CFU/mL of each strain in the cell. The asterisk (^*^) and (^**^) represent statistical significant difference with *P* ≤ 0.05 and *P* ≤ 0.01 comparing with *C. jejuni* RM1221, respectively.

The intracellular survivability of the three strains (1442, 1622, and 1655) was shown in Figure 5. Comparing with RM1221, three selected strains showed remarkable advantage in intracellular survivability in murine macrophage RAW 264.7. RM1221 had short survival time (24 h), while three strains were able to survive for more than 48 h, although a considerable decrease in the number of all internalized was observed. Among them, 1622 and 1655 exhibited highest survivability at 72 h. At each time point, the post-infection number of surviving 1655 was higher than 1622, suggesting that 1655 strain had strongest survivability.

### Motility of the three selected *C. jejuni*

**Figure 6:**
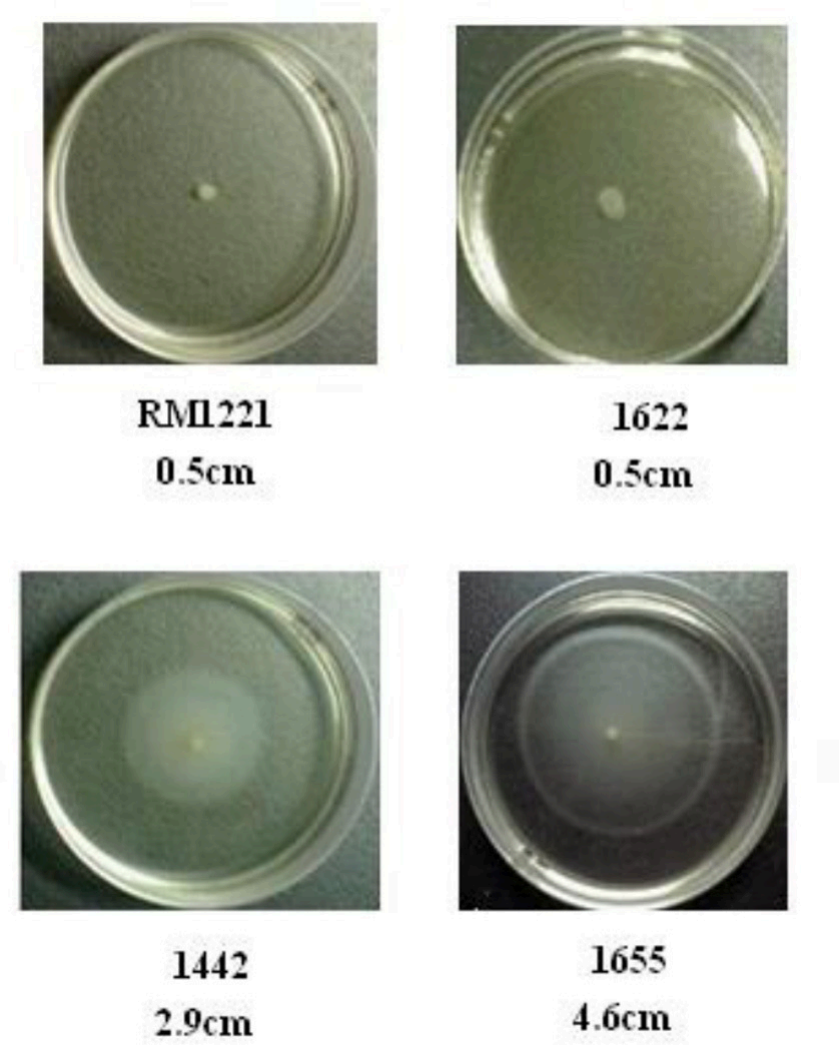
**Motility of the three selected *C. jejuni* strains on 0.4% MH plate**. The motility is positively corrleated to the diameter of growth ring of each strain. The diameter of spotted colonies was 0.5 cm.

As shown in Figure 6, there was apparent difference between diameter growth rings of the three strains (1442, 1622, and 1655) on 0.4% MH agar plate. RM1221 and 1622 were not motile, while 1655 and 1442 had a strong motility.

### Whole-genome features of *C. jejuni* 1655

Based on the above *in vitro* and *in vivo* studies, 1655 was selected for subsequent whole genome sequencing to analyze the potential genetic mechanisms involved in the multidrug resistance and high virulence.

The basic genome information of 1655 and other three reference strains (RM1221, 81–176, and NCTC11168) are summarized into Table 3. The 1655 genome was comprised of a chromosome and a tetracycline resistance plasmid pTet. The pTet plasmid of 1655 had 98% homology with pTet plasmid in 81176. The genome of 1655 was 1720, 061 bp long and contained 1733 predicted coding regions. The genomes of two chicken original strains (1655 and RM1221) were significantly larger than that of two human isolates (*C.* 81–176 and NCTC11168). The GC content of 1655 was 31.36%, consistent with the reference genome sequences.

**Table 3:** **Genomic information of target bacteria 1655 and reference strains**.

Genomic contents	1655	NCTC11168	RM1221	81–176
Refseq number		NC_002163.1	NC_003912.7	NC_008787
Homology (%)	–	88.78	90	90.44
Genome size (bp)	1720061	1641481	1777831	1616554
G+C (%)	31.36	30.5	30.31	30.6
Predicted coding region	1733	–	1898	1770
CDS	1732	1668	1783	1680
Proteins	–	1572	1783	1449
Pseudo gene	323	40	58	35
rRNA	9	9	9	9
tRNA	40	43	44	44
Other RNA	326	4	4	2

**Figure 7:**
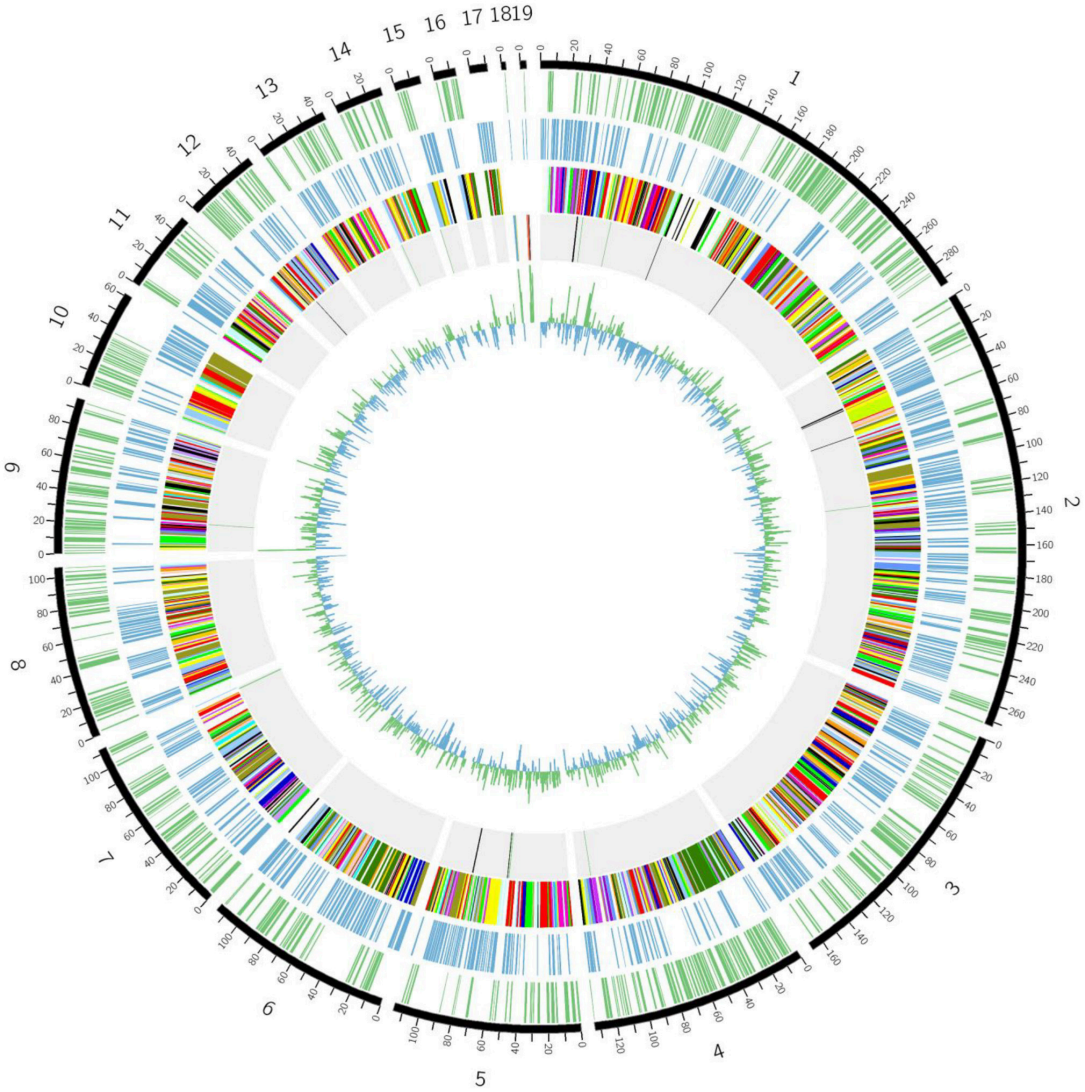
**Schematic circular genome of *C. jejuni* 1655 strain**. From outside to inside, there were five rings. The first circle shows the positive strand genes. The second circle shows the negative strand genes. The third circle were COG functional annotations (chr1-RNA processing and modification; chr2-Chromatin structure and dynamics; chr3-Energy production and conversion; chr4-Cell cycle control, cell division, chromosome partitioning; chr5-Amino acid transport and metabolism; chr6-Nucleotide transport and metabolism; chr7-Carbohydrate transport and metabolism; chr8-Coenzyme transport and metabolism; chr9-Lipid transport and metabolism; chr10-Translation, ribosomal structure and biogenesis; chr11-Transcription; chr12-Replication, recombination and repair; chr13- Cell wall/membrane/envelope biogenesis; chr14-Cell motility; chr15-Posttranslational modification, protein turnover, chaperones; chr16-Inorganic ion transport and metabolism; chr17-Secondary metabolites biosynthesis, transport and catabolism; chr18-General function prediction only; chr19-Signal transduction mechanisms). The fourth circle are rRNA and tRNA (red-16s rRNA, blue-23S rRNA, yellow-5S rRNA, black-positive chain tRNA, green-negative chain tRNA). The fifth circle is the GC contents.

The genome sequence of 1655 was composed by 35 large contigs (Figure 7). The cluster of orthologous groups (COG) assignment for predicted gene products were shown in the third circle from the outside in Figure 7. A total of 1733 putative gene products were assigned to COG identifications classified into 19 COG categories. Three copies of rRNA gene cluster (5s rRNA, 16s rRNA, and 23s rRNA) and 40 tRNA genes were identified on the *C. jejuni* 1655 chromosome.

**Figure 8:**
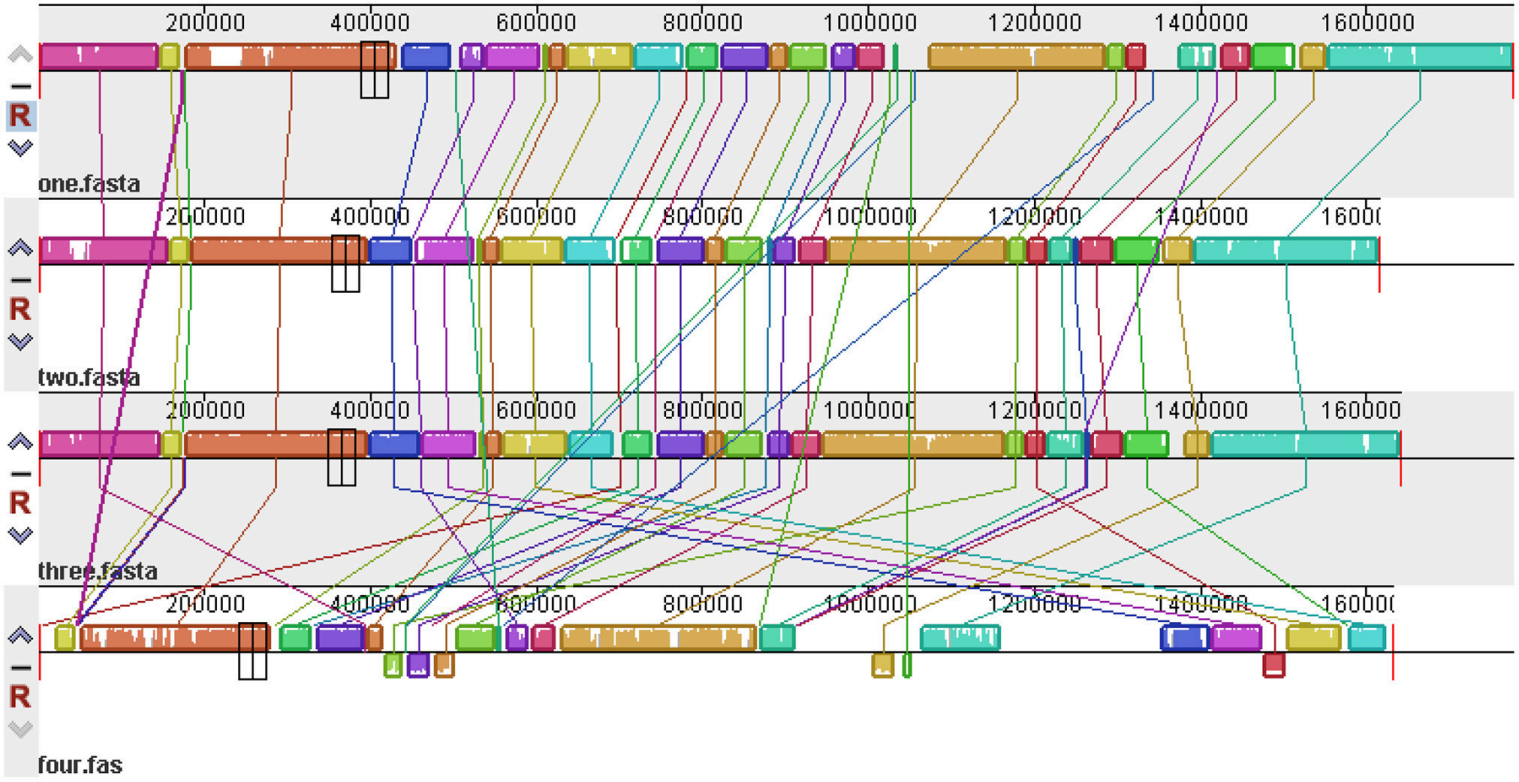
**Global multiple alignment of *C. jejuni* genomes**. From top to bottom are the genome structures of *C. jejuni* RM1221, 81–176, NCTC11168, and 1655, respectively. The color region and white region represented high and low similarity respectively. Above axis was the positive strand, below was the negative strand.

The global alignment of four genomes (RM1221, 81–176, NCTC11168, and 1655) is shown in Figure 8. High similarity was observed in the genome of three reference strains (RM1221, 81–176, and NCTC11168). However, disordered distribution, deletions and rearrangements were observed in the genome of 1655 when compared with genome of three reference strains.

### Resistance determinants in *C. jejuni* 1655

The genome sequencing data showed that 1655 contained T86I mutation in the *gyrA* gene. Some mutations (e.g., A2075G mutation, C2113T, position 1732–1791 mutations) were found in the 23S rRNA of *C. jejuni* 1655. The ribosomal L4 contained V196A mutation and ribosomal L22 had A74G mutation. The tetracycline resistant gene *tetO* was located in pTet plasmid. The *C. jejuni* 1655 also contained *aphA* and *aadE* exogenous genes which mediated resistance to aminoglycosides. No mutation wad found in the regulators (CmeR and CosR) of CmeABC efflux pumps.

### Virulence associated genes in *C. jejuni* 1655

A large number of virulence associated genes were found in the genome of 1655 (Table 4). Among the selected genes involved in flagella synthesis and assembly (*flaA, flaC, flgA, flgB, flhB, fliM, and fliY*), the *flaC* was found in the genome of 1655 and 11168. Two genes (*ciaB* and *nlpC*) associated with invasion and three genes (*cadF, galE*, and *peb2*) related to adhesion were present in 1655 but not in RM1221. Compared to the genes of CPS biosynthesis system in RM1221, the genome of 1655 contained a restriction modification enzyme, sugar nucleotidyltransferase, capsule polysaccharide protein, and alginate O-acetyltransferase. The genome of 1655 also contained some genes (*cfrA* and ExbB-ExbD-TonB) involved in iron uptake system, while RM1221 lacked TonB and 81176 lacked *cfrA* and TonB13. The IS606 transposase, ISHa1675 transposase B and prophage Lp2 protein 6 were also found in the genome of 1655. ISHa1675 transposase B was not found in the genome of three reference strains. The sequence of prophage Lp2 protein in *C. jejuni* 1655 had 99% homology to that in 81–176. The pVir plasmid which previously found in 81–176, was not found in 1655. Three predicted genes of Clustered Regularly Interspaced Short Palindromic Repeats (CRISPR)-associated system were found in the genome of 1655. They may encode Csn1, Cas1, and Cas2 proteins respectively.

**Table 4:** **Virulence relative factors in *C. jejuni* 1655 and reference strains**.

Virulence genes	1655	1221	11168	81176
Virulence	Relative high	No	Low	Intermediate
pVir Plasmid	−	−	−	+
**FLAGELLA SYNTHESIS GENES**				
*flaA*	+	+	+	+
*flaC*	+	−	+	−
*flhB*	+	+	+	+
*flgB*	+	+	+	+
*fliM*	+	+	+	+
*fliY*	+	+	+	+
**INVASION**				
*ciaB*	+	−	+	−
*nlpC*	+	−	−	+
**ADHESION**				
*cadF*	+	−	+	−
*galE*	+	−	−	−
*Peb1*	+	+	+	+
*Peb2*	+	−	+	−
**CHEMOTAXIS**				
*cheY*	+	+	+	+
*motB*	+	+	+	+
*dnaJ*	+	+	+	+
**CPS BIOSYNTHESIS SYSTEM**				
Restriction modification enzyme	+	−	+	−
LPS biosynthesis protein	−	+	−	+
Sugar nucleotidyltransferase (CPS)	+	−	+	+
Capsule polysaccharide protein (KPS)	+	−	+	−
Alginate O-acetyltransferase AlgI	+	−	−	−
**IRON UPTAKE SYSTEM**				
*cfrA*	+	+	+	−
TonB123	+	−	+	−
ExbB	+	+	+	+
ExbD	+	+	+	+
**TRANSPOSASE AND PHAGES PROTEINS**				
IS606 transposase	+	+	+	−
ISHa1675 transposase B	+	−	−	−
Prophage Lp2 protein 6	+	−	−	+
**CRISPR-CAS SYSTEM**				
CRISPR	*csn1-cas1-cas2*	*csn1-cas1-cas2*	*csn1-cas1-cas2-CRISPR-cstIII*	*cstII*

## Discussion

Compared to the genomes of reference strains (NCTC11168, 81–176, RM1221), the genome of 1655 contained some gene inversion, disordered distribution, deletions and rearrangements. The genome difference may be attributed to their different biological characteristics and different evolutional environment. NCTC11168 was originally isolated from a case of human enteritis (Parkhill et al., 2000). 81–176 was originally isolated from a diarrheal outbreak associated with raw milk consumption and exhibited high invasion and high pathogenicity for monkeys and humans (Korlath et al., 1985; Black et al., 1988; Tribble et al., 2010). *C. jejuni* RM1221 (ATCC BAA-1032) was isolated from chicken and showed low virulence (Fouts et al., 2005). 1655 was a multidrug resistant strain isolated from chicken fecal samples that exhibited relative higher virulence, as evidence by its higher pathogenicity to chickens, colonization of the chicken intestinal tract, cytotoxin production, biofilm formation, adhesion/invasion/intracellular survivability to macrophage cell and higher motility.

1655 had multidrug resistance to fluoroquinolones, macrolides, tetracyclines and aminoglycosides. The single T86I mutation in *gyrA* gene contributed to its high-level resistance to FQs (Luangtongkum et al., 2009). High level macrolide resistance in *C. jejuni* 1655 was likely due to A2075G mutation in 23S rRNA (Hao et al., 2009; Luangtongkum et al., 2009; Hao et al., 2010). Although there were some other mutations and deletions in *gyrB*, 23s rRNA genes and ribosomal L4/L22, the function of these mutations on antimicrobial resistance was not evident. The high-level resistance to tetracycline was mediated by *tetO* gene which was located in pTet plasmid (Avrain et al., 2004). The pTet plasmid in 1655 was identical to the one found in 81–176 (Bacon et al., 2002). As a mobile gene element, pTet plasmid may act as a vehicle to pick up and spread multiple antibiotic resistance genes and virulence genes in *C. jejuni* (chen et al., 2013). The *aadE* and *aphA-3* genes in 1655 could explain its resistance to amikacin and gentamicin (Gibreel et al., 2004; Iovine, 2013).

Generally it has been through that multidrug resistance is often associated with higher fitness costs or less virulence (Luangtongkum et al., 2009). However, our study suggested that the multidrug resistant 1655 had higher virulence than the reference strain RM1221 that is not multidrug resistant. Previous studies indicated that T86I mutation in *gyrA* gene could affect DNA supercoiling to regulate the expression of genes associated with bacterial fitness, and therefore enhance the fitness of fluoroquinolone-resistant *Campylobacter* in chicken host and increase the severity and duration of *Campylobacterosis* (Nelson et al., 2004; Luo et al., 2005; Helms et al., 2005; Evans et al., 2009; Han et al., 2012). However, mutations in 23S rRNA could lead to fitness cost and reduced virulence of macrolide resistant *Campylobacter* (Hao et al., 2009; Almofti et al., 2011a,b; Hao et al., 2013). Some of the clinical investigation data indicated the positive correlation between virulence and resistance, but others indicated the negative correlation (Bagger-Skjøt et al., 2007; McGowan-Spicer et al., 2008). For example, two recent studies showed that prevalence of resistance to both erythromycin and ciprofloxacin was higher in isolates harboring *ciaB* and some virulence genes (e.g., *cdtA* and *dnaJ*) were associated with antimicrobial-resistant Campylobacter strains (Ghunaim et al., 2015; Lapierre et al., 2016). Our study revealed the relationship between multidrug-resistance and virulence of some *C. jejuni* chicken isolates, indicating that complex mechanisms may work together to cause multidrug resistance and the relative higher virulence in *C. jejuni 1655*.

Comparing to the genome of RM1221, 1655 contained some special virulence-associated genes and proteins, including *flaC, ciaB, nlpC, cadF, gelE, peb2*, restriction modification enzyme, sugar nucleotidyltransferase, capsule polysaccharide protein, alginate O-acetyltransferase and TonB iron uptake system. The *flaC* has been shown to modulate immune response in the intestinal tract and contribute to bacterial persistence of *C. jejuni* (Faber et al., 2015). The presence of *ciaB*, a *Campylobacter* invasion antigen, may contribute to the high invasion of *C. jejuni* 1655 (Rivera-Amill and Konkel, 1999). The *nlpC* may play a critical role in initial infection process, adherence to host cells (Padhi et al., 2016). The *cadF*, encoding fibronectin protein, may play an important role on the adhesion and colonization of 1655 in chicken (Konkel et al., 1997; Monteville et al., 2003). The *peb2* is a membrane associated protein which may contribute to the increased gastrointestinal virulence of *C. jejuni* (Cordwell et al., 2008). The capsule polysaccharide (CPS), lipooligosaccharide (LOS), and restriction-modification (R/M) systems may enhance the invasion and colonization of 1655 in reservoir hosts and mediate the virulence of 1655 by evading host immune response (Bacon et al., 2001; Suerbaum et al., 2001; Ahmed et al., 2002; Guerry et al., 2002; Klena et al., 2004; Poly et al., 2004; Fouts et al., 2005; Guerry and Szymanski, 2008). The outer membrane protein CfrA and energy transporter system TonB-ExbB-ExbD are associated with iron absorption and iron uptake in gram-negative bacteria (Krewulak and Vogel, 2011). The presence of CfrA and TonB-ExbB-ExbD system in 1655 may contribute to its stronger ability of nutrients uptake and higher intracellular survivability (Andrews et al., 2003; Hofreuter et al., 2006; Krewulak and Vogel, 2011).

The genes encoding IS606 transposase, ISHa1675 transposase B and prophage Lp2 protein 6 were identified in the genome of 1655, suggesting that 1655 may contain transposons or phages. However, future experiments are needed to determine if these are associated with active mobile genetic elements in 1655. The ISHa1675 transposase B and prophage Lp2 protein 6 were not found in the genome of RM1221 and 11168, indicating that these two genes may play special role in the physiological characteristic of *C. jejuni* 1655.

Three proteins associated with CRISPR-Cas systems (CRISPR-Cas2, CRISPR-Cas1 and a hypothetical protein associated with the CRISPR) were found in 1655. The CRISPR-Cas system provides bacterial defense against foreign nucleic acids derived from bacteriophages or plasmids (Barrangou et al., 2007; Marraffini, 2013). It also plays an important role in gene regulation and bacterial pathogenicity (Mojica et al., 2005; Louwen et al., 2013; Louwen and van Baarlen, 2013; Sampson and Weiss, 2014). Strains containing CRISPR-Cas system have a stronger ability of biofilm formation and colonization in mouse organs than those strains lack this system (Shimomura et al., 2011). Therefore, the presence of CRISPR-Cas system may contribute to the high virulence and biofilm formation capacity of *C. jejuni* 1655 strain.

## Conclusion

1655 strain exhibited multidrug resistance to fluoroquinolone, macrolide, tetracycline, and aminoglycoside drugs and relative high *in vitro* and *in vivo* virulence. Comparing to the genome of reference strains (NCTC11168, RM1221, and 81–176), there were large difference in the genome structure and genome content in 1655. The co-emergence of target gene mutations, resistance genes and pTet plasmid could explain its multidrug resistance. The virulence mechanism of *Campylobacter* may be mediated by a variety of virulence factors, including proteins involved in flagella biosynthesis, invasion, adhesion, CPS biosynthesis system, iron uptake system, transposase, phage proteins, and CRISPR-Cas system. Future experiments are needed to find the deeper molecular mechanisms and confirm the function of some important gene elements on multidrug resistance and relative higher virulence.

## Financial disclosure

This work was supported by National Basic Research Program of China (2013CB127200), National Key research and development program (2016YFD0501302), National Natural Science Foundation of China (31101856), National Key Technology R&D Program (2012BAK01B00), Morning program of Wuhan in China (2015070404010191), Fundamental Research Funds for the Central Universities (2662015PY035), and National Program for Risk Assessment of Quality and Safety of Livestock and Poultry Products (GJFP2016008). The funders had no role in study design, data collection and analysis, decision to publish, or preparation of the manuscript. The opinions expressed in this manuscript are solely the responsibility of the authors and do not necessarily represent the official views and policy of the US Food and Drug Administration or National Institutes of Health. Reference to any commercial materials, equipment, or process does not in any way constitute approval, endorsement, or recommendation by the Food and Drug Administration.

## Author contributions

Conceived and designed the experiments: HH, NR, MD, GC, ZY. Performed the experiments: NR, XK, JL, ZI, HH. Analyzed the data: HH, NR, JH, SF, YW, ZY. Contributed reagents/materials/analysis tools: YW, ZL, MD, YW, ZY. Wrote the paper: HH, NR, JH, SF, ZI, ZY.

### Conflict of interest statement

The authors declare that the research was conducted in the absence of any commercial or financial relationships that could be construed as a potential conflict of interest.
