# Supplementary material for: Virulence and Genomic Feature of Multidrug Resistant Campylobacter jejuni Isolated from Broiler Chicken
Source: Front Microbiol. 2016 Oct 14;7:1605. doi: 10.3389/fmicb.2016.01605 (PMC5064177; doi:10.3389/fmicb.2016.01605)
Supplement: Supplementary file 1 [file Table1.docx]

**Supplementary data**

Table S1. Pathological change after oral inoculation by 10^5^ CFU of each *C. jejuni*

| Strain | Clinical features | Duration |
| --- | --- | --- |
| **RM1221** | Diarrhea, bloody stools | Recovered at day 4 |
| 1442 | No observed clinical change |  |
| 1447 | No observed clinical change |  |
| 1614 | Diarrhea, bloody stools | Recovered at day 4 |
| 1622 | Diarrhea, bloody stools | Recovered at day 8 |
| 1655 | Diarrhea, bloody stools | Recovered at day 10 |
| 1685 | Diarrhea, bloody stools | Recovered at day 4 |
| Negative control | Healthy, normal |  |
